# Dark-blood late gadolinium enhancement without additional magnetization preparation

**DOI:** 10.1186/s12968-017-0372-4

**Published:** 2017-08-23

**Authors:** Robert J. Holtackers, Amedeo Chiribiri, Torben Schneider, David M. Higgins, René M. Botnar

**Affiliations:** 1 Division of Imaging Sciences and Biomedical Engineering, King’s College London, London, United Kingdom; 2 Department of Radiology, Maastricht University Medical Centre, Maastricht, the Netherlands; 3 Philips, Guildford, Surrey, United Kingdom; 4 Pontificia Universidad Católica de Chile, Escuela de Ingeniería, Santiago, Chile

**Keywords:** Delayed enhancement, Late enhancement, Late gadolinium enhancement, Dark blood, Phase-sensitive inversion-recovery, Myocardial infarction, Myocardial scar

## Abstract

**Background:**

This study evaluates a novel dark-blood late gadolinium enhancement (LGE) cardiovascular magnetic resonance imaging (CMR) method, without using additional magnetization preparation, and compares it to conventional bright-blood LGE, for the detection of ischaemic myocardial scar. LGE is able to clearly depict myocardial infarction and macroscopic scarring from viable myocardium. However, due to the bright signal of adjacent left ventricular blood, the apparent volume of scar tissue can be significantly reduced, or even completely obscured. In addition, blood pool signal can mimic scar tissue and lead to false positive observations. Simply nulling the blood magnetization by choosing shorter inversion times, leads to a negative viable myocardium signal that appears equally as bright as scar due to the magnitude image reconstruction. However, by combining blood magnetization nulling with the extended grayscale range of phase-sensitive inversion-recovery (PSIR), a darker blood signal can be achieved whilst a dark myocardium and bright scar signal is preserved.

**Methods:**

LGE was performed in nine male patients (63 ± 11y) using a PSIR pulse sequence, with both conventional viable myocardium nulling and left ventricular blood nulling, in a randomized order. Regions of interest were drawn in the left ventricular blood, viable myocardium, and scar tissue, to assess contrast-to-noise ratios. Maximum scar transmurality, scar size, circumferential scar angle, and a confidence score for scar detection and maximum transmurality were also assessed. Bloch simulations were performed to simulate the magnetization levels of the left ventricular blood, viable myocardium, and scar tissue.

**Results:**

Average scar-to-blood contrast was significantly (*p* < 0.001) increased by 99% when nulling left ventricular blood instead of viable myocardium, while scar-to-myocardium contrast was maintained. Nulling left ventricular blood also led to significantly (*p* = 0.038) higher expert confidence in scar detection and maximum transmurality. No significant changes were found in scar transmurality (*p* = 0.317), normalized scar size (*p* = 0.054), and circumferential scar angle (*p* = 0.117).

**Conclusions:**

Nulling left ventricular blood magnetization for PSIR LGE leads to improved scar-to-blood contrast and increased expert confidence in scar detection and scar transmurality. As no additional magnetization preparation is used, clinical application on current MR systems is readily available without the need for extensive optimizations, software modifications, and/or additional training.

## Background

In the past decade, cardiovascular disease death rates have seen a strong decline, mainly due to cardiovascular risk factor control intervention. However, approximately one of every seven deaths in the United States is still caused by coronary heart disease [1]. As survivors of myocardial infarction face a substantially higher risk of new cardiovascular events due to heart failure, accurate diagnosis and guidance of treatment are crucial in these patients. An accurate assessment of the extent and transmurality of the irreversibly injured cardiac tissue (scar tissue) is essential information in the identification of patients at increased risk of future events and in the selection of the best therapeutic approach. Even tiny regions of scar tissue of only 2% of the mean left ventricular (LV) mass are linked with a sevenfold increase in major cardiac events [2]. Transmurality of the affected area is equally of great importance, as it plays a major role in the prediction of the likelihood of regional functional recovery after revascularization [3–6].

Late gadolinium enhancement (LGE) cardiovascular magnetic resonance imaging (CMR) has been considered the reference standard in the non-invasive assessment of myocardial viability for almost two decades, as LGE is able to clearly depict myocardial infarction and macroscopic scarring from viable myocardium. LGE is based on an inversion-recovery (IR) pulse sequence that is performed 10–20 min after the intravenous injection of a gadolinium-based contrast agent. This pulse sequence is highly T_1_-weighted and therefore sensitive for detecting the local effects of high concentrations of gadolinium. The pulse sequence consists of a 180° inversion pulse, followed by a delay time (called inversion time or TI), and finally signal acquisition. The duration of the TI can be adjusted to minimize or even null the magnetization, which is routinely used in clinical practice to eliminate signal from viable myocardium. However, due to the bright signal of adjacent LV blood, the border between scar and blood can be difficult to identify and the apparent volume of scar tissue can be significantly reduced, or even completely obscured. Obscuration particularly occurs in cases of thin subendocardial scarring, mostly caused by coronary artery disease and subsequent myocardial infarction. In addition, blood pool signal can mimic scar tissue and lead to false positive observations. Simply shortening the TI to null the LV blood magnetization not only results in a negative signal of the viable myocardium, which appears equally as bright as scar due to the magnitude image reconstruction, but also leads to significantly smaller scar size [7, 8]. Furthermore, heart rate variations during acquisition often result in suboptimal TIs for tissue nulling and image artefacts.

**Fig. 1:**
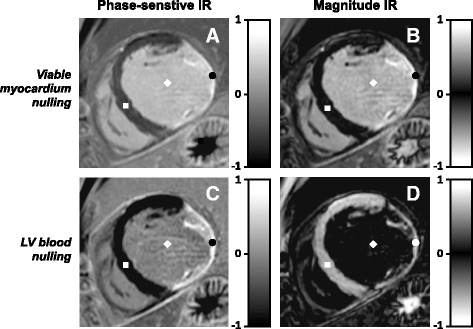
Phase-sensitive inversion-recovery (PSIR) and magnitude IR late gadolinium enhancement (LGE) images with viable myocardium nulling and blood nulling. Routine viable myocardium nulling is shown in **a** & **b**, while the proposed left ventricular (LV) blood nulling is shown in **c** & **d**. The viable myocardium, LV blood, and scar tissue are indicated by a ■, ♦, and •, respectively. Note that the nulled viable myocardium appears *black* using magnitude IR (in **b**), whereas it appears *dark gray* using PSIR (in **a**). The corresponding *gray scale bars* for magnitude IR and PSIR are shown on the right of each image

These problems can be mitigated using phase-sensitive inversion-recovery (PSIR). PSIR is commonly used for LGE image acquisition as it avoids the need for precise selection of the TI to null viable myocardium [7]. Compared to a conventional IR sequence, the PSIR sequence only applies a 180° inversion pulse once every two heartbeats and a small-flip-angle reference acquisition is performed during the second heartbeat. This reference acquisition is used to accurately determine the phase of the measured signal, acquired during the first heartbeat. The PSIR sequence is therefore able to distinguish between positive and negative longitudinal magnetization and will represent the recovered longitudinal magnetization (M_z_) differently in the (corrected real) image produced for clinical assessment: negative M_z_ appears darkest, nulled tissue appears mid-gray, and positive M_z_ appears bright; whereas in a traditional magnitude image nulled tissue appears darkest and both negative and positive M_z_ appear bright (Fig. 1). PSIR is routinely used in combination with nulling of the viable myocardium magnetization, and the clinical observer may adjust window levels to further darken viable myocardial tissue, mimicking a magnitude image representation while maintaining the benefits of PSIR.

We propose a PSIR-specific TI optimization that instead nulls blood and thus significantly improves scar-to-blood contrast while maintaining the scar-to-myocardium contrast. As no additional magnetization preparation is used, clinical application is readily available on current MR systems without the need for extensive optimizations, software modifications, and/or additional training.

PSIR’s extended grayscale range provides an opportunity to achieve a darker blood signal whilst preserving bright scar and dark myocardium signal. Setting a shorter TI, such that the LV blood magnetization is close to the null point of recovery, leads to a darker-gray appearance of the LV blood in the PSIR image. The negative magnetization from viable myocardium, that would appear bright in a magnitude image, now appears completely black, as PSIR reveals its negative phase. The positive scar magnetization still appears bright due to its fast recovery, leading to an increased dynamic contrast range and therefore offering excellent contrast between areas of scar and both LV blood and viable myocardium (Fig. 1c).

## Methods

### Study population

Patients enrolled for a clinical LGE MRI examination at the St. Thomas Hospital in London (Guy’s and St Thomas’ NHS Trust) were eligible to participate if they were >18 years of age and agreed to 15 min of additional MR imaging after the clinical protocol. Only patients showing ischemic myocardial scar on the clinical images were included and underwent additional MR imaging. Patients unable to hold their breath or with poor vectorcardiogram signal detection during the additional imaging were excluded from analysis. Participants provided written informed consent for the study inclusion and additional imaging. The study was conducted according to the Declaration of Helsinki and Good Clinical Practice guidelines and was approved by the local ethics committee (ethics approval number 15/NS/0030).

### MR imaging

MR imaging was performed using a 1.5 T whole-body MR system (Ingenia; Philips, The Netherlands) with dedicated anterior and posterior torso receiver coils. As part of the clinical protocol, conventional LGE was performed by acquiring a stack of short-axis and multiple long-axis views in each patient. These clinical LGE images were acquired 10–15 min after intravenous injection of 0.2 mmol/kg gadobutrol (Gadovist; Bayer, Germany) using a PSIR turbo field echo pulse sequence (echo time (TE) 3.0 ms, repetition time (TR) 6.1 ms, flip angle 25°, reference flip angle 5°, 19 lines acquired every other RR-interval, linear profile order, field of view 350x350mm, slice thickness 10 mm, acquisition matrix 220 × 170, reconstructed voxel size 0.91 × 0.91 mm) during the mid-diastolic resting period. All images were acquired during repetitive 10–15 s breath-holds. A preceding Look-Locker sequence (TI scout) was performed to determine the correct inversion time for viable myocardium nulling.

In case participating patients showed ischemic myocardial infarction in (one of) the acquired clinical images, additional MR imaging was performed in a single slice short-axis view showing the highest scar burden. A PSIR turbo field echo pulse sequence (identical to the clinical one described above) was performed twice with different TIs set for LV blood nulling and viable myocardium nulling in a randomized order for each patient. Both pulse sequences were performed during 10–15 s breath-holds. A preceding Look-Locker sequence was performed for each pulse sequence to determine up-to-date TIs for both LV blood nulling as well as viable myocardium nulling. A dedicated noise scan (identical pulse sequence without excitation pulses) was performed afterwards to assess the noise level. Additionally, in some patients a modified Look-Locker inversion-recovery (MOLLI) T_1_-mapping scan (3–5 scheme) was performed to determine T_1_ values of the viable myocardium, LV blood, and scar tissue [9].

### SNR and CNR measurements

For all acquired images, the manufacturer’s applied scaling in the stored DICOM data was removed by converting the data to floating point values as this reflects the true MR signal range directly after reconstruction [10]. An expert observer (AC) with >10 years of experience in cardiovascular MRI then manually drew regions of interest (ROIs) in the viable myocardium, LV blood pool, and scar tissue, while blinded to patient and image type, using a custom-made MATLAB (The Mathworks, Natick, MA, USA) software tool. For each PSIR image, the observer was able to set image contrast (window level and window width) as preferred, while the image intensity histogram was shown. Finally, the outer border of the entire LV was delineated.

Within each of the three tissue ROIs in each PSIR image, the mean signal intensity was calculated as a measure of the signal level. The entire LV ROI was then projected onto the noise scan image. Within this ROI, the standard deviation of the noise signal intensity was calculated as a measure of the LV noise level. This measure was used for both PSIR images in each patient, as noise was not expected to vary at different inversion times. In each PSIR image, the signal-to-noise ratio (SNR) in the three tissues of interest was calculated by dividing the tissue’s signal level by the LV noise level. Contrast-to-noise ratios (CNRs) between all three tissues were calculated, for both LV blood nulling as well as viable myocardium nulling, by subtracting the SNRs of two corresponding tissues.

### Expert analysis

The same expert observer also 1) estimated the maximum scar transmurality, 2) assigned a confidence score for scar detection and maximum transmurality, 3) traced the scar border to accurately assess scar size, and 4) indicated the circumferential endpoints of the scar to calculate the angle over which the scar was present. First, maximum scar transmurality was estimated using a 5-point scale, as follows: 1 = 0%–20%, 2 = 21%–40%, 3 = 41%–60%, 4 = 61%–80%, and 5 = 81%–100%. Second, confidence in scar detection and maximum transmurality was scored using a 4-point Likert scale, as follows: 1 = non-diagnostic, 2 = low, 3 = medium, 4 = high. Third, scar size was assessed as the total number of voxels within each traced scar area. Differences in scar size (between the two nulling times) were normalized using the scar size acquired with viable myocardium nulling. Enhancement of the papillary muscle was not included in this analysis. Finally, the circumferential angle of the present scar was calculated by indicating the LV centre point, as well as both circumferential scar endpoints in the myocardial wall.

### Statistical analysis

The calculated CNRs in the PSIR images acquired at viable myocardium nulling were compared to those acquired at LV blood nulling. Paired-sample t-tests were used to test for significant differences in CNR at the two different nulling times. For the expert analyses, Wilcoxon signed-rank tests were used to test for significant differences in maximum transmurality and confidence scores, while a one-sample t-test was used to test for significant differences in normalized scar size. A paired-sample t-test was used to test for significant differences in circumferential scar angle. For the paired-sample t-tests and one-sample t-test, normality of the data was confirmed by using Shapiro-Wilk tests. *P*-values <0.05 were considered significant.

### Simulations

Bloch simulations were performed in MATLAB to simulate the magnetization levels of the viable myocardium, LV blood, and scar tissue, over multiple heartbeats. The simulations included all sequence parameters and pulse sequence details, such as start-up echoes, flip angle sweeps, transverse signal spoiling, and image and PSIR reference acquisitions. The relaxation times of the viable myocardium, LV blood, and scar tissue, were determined using the acquired T_1_ maps. Other relevant patient specific model parameters, such as heart rate, trigger delay, and inversion times, were based on the actual scan parameters.

## Results

### Study population

**Fig. 2:**
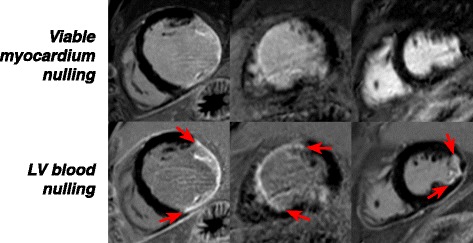
Phase-sensitive inversion-recovery (PSIR) late gadolinium enhancement (LGE) images in three myocardial infarction patients with ischemic scar. These images are acquired with routine viable myocardium nulling (*top row*) and proposed left ventricular (LV) blood nulling (*bottom row*). Note that all images are windowed so that viable myocardium appears homogeneously black. The *arrows* indicate the circumferential borders of the LV scar tissue

Of all patients who provided written informed consent, nine male patients (63 ± 11y) showed ischemic myocardial scar and underwent additional imaging. Imaging at both inversion times, LV blood nulling and viable myocardium nulling, was successfully performed at 22 ± 4.6 min and 23 ± 6.2 min post-injection, respectively (Fig. 2). These post-injection times were not significantly different (*p* = 0.220). The inversion times used for LV blood nulling and viable myocardium nulling were 168 ± 28 ms and 259 ± 25 ms, respectively.

### SNR and CNR measurements

**Fig. 3:**
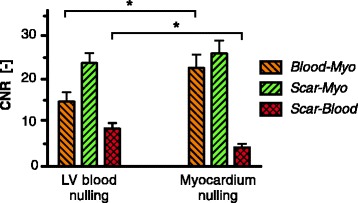
Mean contrast-to-noise ratios (CNRs) between the viable myocardium, left ventricular (LV) blood, and scar tissue. The CNRs are shown for both LV blood nulling and viable myocardium nulling. *Error bars* indicate the standard error of the mean. * = *p* < 0.001

**Fig. 4:**
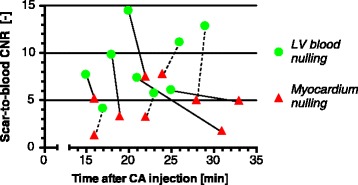
Scar-to-blood contrast-to-noise ratios (CNRs) with their associated elapsed time after contrast agent (CA) injection. The *dots* indicate ventricular (LV) blood nulling, while the *triangles* indicate viable myocardium nulling. For each subject, a *straight line* connects the two observations. Note that a *dotted line* indicates that viable myocardium nulling was performed first, while a *solid line* indicated that LV blood nulling was performed first

The average scar-to-blood CNR in the PSIR images was significantly increased (*p* < 0.001) by 99% to 8.78 when nulling LV blood magnetization instead of viable myocardium (Fig. 3). A significant mean difference of +4.37 (95% CI [+2.53, +6.22]) was observed in scar-to-blood CNR when nulling LV blood magnetization. This increase in scar-to-blood CNR was observed in all patients regardless of which nulling time was used first (Fig. 4). Despite the significant increase in scar-to-blood CNR when nulling LV blood, a significant change in average scar-to-myocardium CNR was not detected (*p* = 0.150). The average blood-to-myocardium CNR, however, was significantly decreased (*p* < 0.001) by 34% when nulling LV blood. The measured SNRs for viable myocardium, LV blood, and scar, for both myocardium nulling and LV blood nulling, can be found in Table 1. The elapsed times after contrast agent administration and used inversion times are also illustrated here, as well as measured T1 values for the three tissues of interest.

**Table 1:** Signal-to-noise-ratios (SNRs), inversion times, and post-injection times for viable myocardium nulling and LV blood nulling

Subject	Viable myocardium nulling					LV blood nulling					MOLLI T1 mapping^a^			
	Time after injection [min]	Inversion time [ms]	Myocardium SNR [−]	Blood SNR [−]	Scar SNR [−]	Time after injection [min]	Inversion time [ms]	Myocardium SNR [−]	Blood SNR [−]	Scar SNR [−]	Time after injection [min]	T1 Myocardium [ms]	T1 Blood [ms]	T1 Scar [ms]
1	16	250	0.56	36.02	37.27	17	140	−19.42	6.22	10.32	21	361	207	175
2	31	262	−0.41	9.12	10.83	21	169	−9.16	−1.45	5.89	-	-	-	-
3	22	239	−4.37	16.88	20.08	23	169	−11.16	0.62	6.33	28	349	271	195
4	16	239	−4.37	26.14	21.00	15	145	−20.13	−1.68	5.99	20	327	235	170
5	28	275	−1.82	19.80	24.78	29	185	−14.19	0.00	12.82	22	390	265	180
6	24	300	−0.09	12.65	20.41	26	220	−8.16	−0.10	11.02	28	430	345	240
7	33	286	−2.76	21.48	26.40	25	187	−14.06	1.85	7.89	26	390	280	205
8	22	220	−5.35	27.32	34.78	20	127	−23.18	−1.04	13.41	-	-	-	-
9	19	261	0.37	16.15	19.40	18	168	−11.29	−0.78	9.01	-	-	-	-
*mean*	*23*	*259*	*−2.03*	*20.62*	*23.88*	*22*	*168*	*−14.53*	*0.40*	*9.19*	*24*	*375*	*267*	*194*
*SD*	*6.2*	*25*	*2.28*	*8.24*	*8.14*	*4.6*	*28*	*5.26*	*2.44*	*2.89*	*4.0*	*36*	*47*	*26*

^a^Note that 3-5 MOLLI T1 mapping was not always successfully performed in each patient

*MOLLI* modified Look-Locker inversion-recovery, *SD* standard deviation, *SNR* signal-to-noise ratio

### Expert analysis

**Fig. 5:**
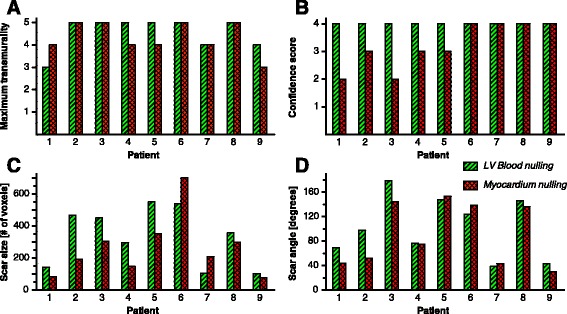
Expert analysis results for maximum scar transmurality (**a**), confidence score for scar detection and maximum transmurality (**b**), scar size (**c**), and longitudinal scar angle (**d**). In all four subplots, the results are shown for each subject for both left ventricular (LV) blood nulling [*diagonally striped* / *green*] and viable myocardium nulling [*checkered* / *red*]

No significant difference in maximum scar transmurality was detected between LV blood nulling and viable myocardium nulling (*p* = 0.317). In three patients, higher maximum scar transmurality was observed when nulling LV blood, while this was only the case in one patient when nulling viable myocardium (Fig. 5a). All observed differences were less than 20%. LV blood nulling, however, did lead to significantly higher confidence scores (*p* = 0.038) compared to nulling viable myocardium. The maximum confidence score of 4 (=high) was assigned to all patients when nulling LV blood, while only four patients received this score when viable myocardium was nulled (Fig. 5b). None of the PSIR images, regardless of nulling time, were assigned with non-diagnostic confidence. No significant difference in normalized scar size was detected between LV blood nulling and viable myocardium nulling (*p* = 0.054). However, in seven patients a larger scar area was observed when nulling LV blood (Fig. 5c). In five of these patients, the observed scar size was more than 40% larger when nulling LV blood compared to nulling viable myocardium. In all these cases a higher confidence score was assigned to LV blood nulling. Finally, no significant difference in circumferential scar angle was detected between LV blood nulling and viable myocardium nulling (*p* = 0.117). On average, the circumferential scar angle was 12 degrees larger when nulling LV blood (Fig. 5d). Differences in scar angle ranged from −14 to +46 degrees when nulling blood compared to viable myocardium.

### Simulations

**Fig. 6:**
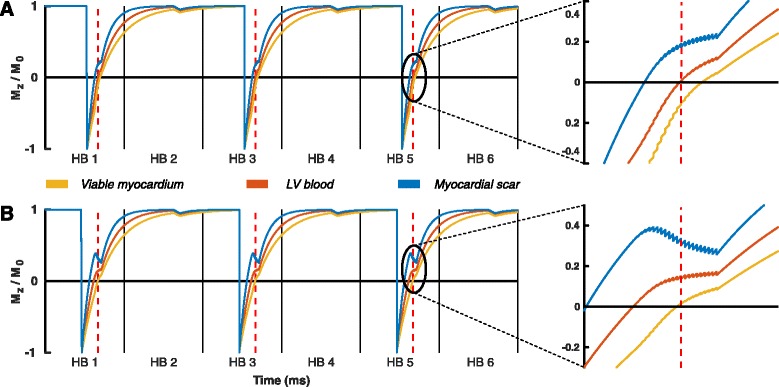
Bloch simulations of the (normalized) longitudinal magnetization (M_z_). The M_z_ of viable myocardium [*yellow*], left ventricular (LV) blood [*orange*], and myocardial scar [*blue*], are shown in a phase-sensitive inversion-recovery (PSIR) pulse sequence with LV blood nulling (**a**) and viable myocardium nulling (**b**). For both subplots, the approach to the steady state during acquisition has been enlarged on the right to show the fine pulse sequence details. The *vertical black solid lines* indicate the end of a heartbeat (HB), while the *vertical red dotted lines* indicate the acquisition of the middle of k-space. Note that the time between the inversion pulse and the *red dotted line*, the inversion time, is significant shorter with LV blood nulling (**a**) than with viable myocardium nulling (**b**)

Bloch simulations of the magnetization levels confirmed the near-perfect nulling of both LV blood as well as viable myocardium when using the corresponding pulse sequence and tissue parameters (Fig. 6). The simulations also illustrated the magnetization evolution in the approach to the steady state during acquisition. These approaches are differently shaped for viable myocardium nulling and LV blood nulling, and therefore contributing to the observed contrast differences. These different shapes are caused by the fact that even though the time between the excitation pulses in the acquisition shot (=TR) is the same for both nulling times, magnetization recovery within each TR is faster when the TI is set for blood nulling as the magnetization is further away from the equilibrium value.

## Discussion

Accurate subendocardial scar assessment in patients with myocardial infarction is crucial as current treatment strategies are based on this assessment and even tiny regions of myocardial scar may already have great impact on patient prognosis. Conventional LGE using viable myocardium magnetization nulling is not always able to detect these tiny regions of scar as the LV blood often has similar signal intensity. Lowering the LV blood signal may be achieved by using additional magnetization preparation before or after the 180° inversion pulse. Multiple types of additional magnetization preparation have recently been proposed, including T_2_ preparation, magnetization transfer, T_1_ rho using spin locking, and double and triple inversion recovery [11–18]. All the above-mentioned magnetization preparation schemes alter magnetization levels, thereby creating different types of contrast and possibly improving scar visibility. However, these magnetization preparations require extensive optimizations, additional training, and are not available in routine clinical practice. Furthermore, specific energy absorption rate (SAR) levels are increased due to the additional radiofrequency pulses.

In this study we presented a PSIR LGE method, without using additional magnetization preparation, which improves subendocardial scar conspicuity. Shortening the TI to the point of LV blood magnetization nulling, in combination with PSIR reconstruction, led to a significant increase in scar-to-blood contrast, while maintaining excellent scar-to-myocardium contrast. Regardless of whether normal viable myocardium nulling or LV blood nulling was performed first, all subjects showed increased scar-to-blood contrast with LV blood nulling. As the washout of the contrast agent is faster in the LV blood pool than in the infarct, scar-to-blood contrast is expected to naturally increase at later imaging time points. However, even though myocardium nulling was performed approximately 10 min later than LV blood nulling in two subjects, scar-to-blood contrast still remained higher with LV blood nulling. The significant loss in blood-to-myocardium contrast does not interfere with scar detection and analysis, as this contrast is only used for anatomical visualisation of the viable myocardium. Moreover, the observed blood-to-myocardium contrast during LV blood nulling was still abundant.

Expert analysis showed that higher levels of confidence were assigned to PSIR images acquired during LV blood nulling compared to viable myocardium nulling. Especially in those patients with lower confidence scores during viable myocardium nulling, large differences in scar size and circumferential scar angle, up to 143% and 87% respectively, were observed when nulling LV blood compared to nulling viable myocardium. Although no significant differences in maximum scar transmurality were detected, expert confidence in the assessed transmurality was similar or higher when LV blood was nulled, in all patients. The assessed scar size was on average 44% larger when nulling LV blood, however this difference was not significant (*p* = 0.054). However, we expect to detect significant differences in scar size when a larger and more diverse patient group is included. Moreover, these images were reviewed by an expert operator with level III accreditation and more than 10 years of experience. The advantage of using dark-blood DE in less experienced operators shall be assessed in future studies.

### Simulations

Our Bloch simulations predict and confirm the nulling of LV blood and viable myocardium when using corresponding pulse sequence and tissue parameters. While differences in steady state behaviour can partially explain the effect on contrast changes, those are difficult to predict precisely, as the observed contrast can depend on numerous other variables such as contrast agent dosage, elapsed time after contrast agent administration, and clearance rate of the contrast agent. Blood velocity may also play a role, even though non-selective IR pulses are used [18]. Furthermore, additional effects, such as unknown levels of off-resonance, magnetization transfer, and insufficiently spoiled T_2_ magnetization, could play a role.

### PSIR use in combination with LV blood nulling

In current clinical practice, wherein viable myocardium is nulled on magnitude images, PSIR is mainly used to make DE image quality less sensitive to the chosen TI, leading to a reduction in image artefacts. However, the main feature of PSIR, which is retaining the sign of the signal intensity, is then not fully exploited, as all magnetization levels are either nulled or already positive. When image acquisition is performed at shorter TIs, for example at LV blood nulling, the main feature of PSIR is optimally exploited resulting in perfect black myocardium, dark gray LV blood at the null point, and bright scar. Already in 2002, Kellman et al. showed that scar size appearance was significantly underestimated when using shorter TIs than the nulling point of viable myocardium when using a traditional magnitude image reconstruction [7]. In 2005, Setser et al. also investigated scar size appearance with conventional IR and found similar outcomes at TIs 50 ms shorter than optimal myocardium nulling, however showed that this underestimation was not observed when using these shorter TIs in combination with PSIR [8]. We found similar results in this study, justifying the use of shorter inversion times in PSIR. In contrast to previous studies, we aimed here explicitly for optimal LV blood nulling, which was achieved at TIs that were on average 91 ms shorter than optimal viable myocardium nulling.

The breath-hold duration of the proposed PSIR sequence with LV blood nulling is equal to the conventional PSIR sequence with viable myocardium nulling, as only the inversion time is shortened. When conventional IR is used instead of PSIR during routine clinical LGE, the breath-hold duration will double to approximately 10–15 s as the 180° inversion pulse is only applied every other heartbeat for PSIR. However, since the PSIR allows additional relaxation of the magnetization levels during the extra, second R-R interval, increased signal levels are achieved resulting in higher SNR. At the expense of this extra SNR, pulse sequence duration may be shortened using parallel imaging methods, such as spatial sensitivity encoding, to compensate for the extra scan time originally required for PSIR.

### Limitations

All patients used in this study were already enrolled for a clinical LGE examination, meaning that additional imaging for this study could only be performed afterwards. Therefore the study images were acquired between approximately 15–30 min post-injection. Even though this is later than what would normally be done in routine clinical care, it still remains within the 10–30 min post-injection time window in which LGE is ideally performed [19]. Acquisition of the study images started immediately when the clinical protocol was finished, after which the available radiologist directly reviewed the quality of the earlier acquired clinical images. In two subjects, additional clinical images needed to be acquired that delayed the acquisition of one of the two study images. This explains the large difference in elapsed time after contrast administration between the two acquisitions in these two subjects.

Two additional topics that require further discussion are the possible effects of low flow regions near the LV endocardium and possible distortions of the infarct edges due to the presence of both positive and negative magnetization in the same voxel. Low flow near the endocardium should not have a significant effect on the observed signal, as the inversion pulse is non-selective and thus should be flow independent. In addition, as can be observed from our simulations in Fig. 6, blood magnetization in the second heartbeat is already fully recovered and close to the equilibrium magnetization before the acquisition of the reference image. Since the blood magnetization is hardly affected by the following low flip angle reference acquisition, the blood magnetization again fully recovers before the next inversion pulse is applied. As a result, regions of slow flow near the LV border do not affect the signal here as they behave similar to the rest of the blood in the LV cavity.

Finally, when using PSIR with LV blood nulling, the TI is set shorter compared to viable myocardium nulling. Therefore the viable myocardium will have negative magnetization while the infarct region already has positive magnetization. Voxels close to the edge of the infarcted region can therefore obtain both positive and negative magnetization levels, possibly leading to cancellation of the resulting magnetization in that voxel and distorting the edge of the infarct. As this voxel behaves similarly to tissue that would be nulled, it appears dark grey in a PSIR image. However, such a voxel would also appear grey when using a conventional IR sequence with viable myocardium nulling, as that voxel holds both positive magnetization from the scar area (bright) and nulled magnetization from the viable myocardium (black in normal IR image). In both situations the scar border appears greyish, which is a natural transition from the bright scar area to the black viable myocardium.

### Future perspectives

This study demonstrates the effect of using shorter TIs in combination with PSIR during 2D LGE for the detection of ischemic myocardial scar. However, the proposed mechanism can also be applied in a free-breathing 3D pulse sequence, offering new opportunities for patients who are short of breath or who cannot cope with long breath-hold durations. For future research, additional studies are required that include a larger number of patients with varying underlying causes of (myocardial) scar. It is expected that significant differences in scar size and transmurality will be found when using a larger and more diverse patient population. Multiple clinical experts should then be included in the analysis of the observed myocardial scar. Finally, automatic detection and quantification of scar areas near the endocardial border is likely to improve as a result of the increased scar-to-blood contrast.

## Conclusions

Nulling viable myocardium for PSIR LGE is a continuation of routine clinical practice from the time non-phase-sensitive (magnitude) images were acquired with LGE. For PSIR LGE, nulling the LV blood magnetization instead significantly increases scar-to-blood contrast since blood signal and scar signal no longer have similar levels. We introduced a novel method that allows visualization of contrast-enhanced tissues, while suppressing the blood, and thereby improving subendocardial scar conspicuity in PSIR LGE. As no additional magnetization preparation is used, clinical application on current MR systems is readily available without the need for extensive optimizations, software modifications, and/or additional training.

## Abbreviations

CNR: Contrast-to-noise ratio
DICOM: Digital imaging and communication in medicine
IR: Inversion-recovery
LGE: Late gadolinium enhancement
LV: Left ventricular
MOLLI: Modified look-locker inversion-recovery
MRI: Magnetic Resonance Imaging
PSIR: Phase-sensitive inversion-recovery
ROI: Region of interest
SAR: Specific absorption rate
SNR: Signal-to-noise ratio
TE: Echo time
TI: Inversion time
TR: Repetition time
